# Poisoning

**Published:** 1879-08

**Authors:** 


Poisoning.—In one dozen cases of poisoning from the bite of the rattlesnake, iodine proved curative, given in one or two drop doses of the tincture every hour, according to the severity of the case. In one instance, where the patient was "swollen terribly, mottled spots appearing over the entire body, breathing with great difficulty, and apparently near death," four drops of iodine were given every hour with entire recovery.—*Dr. E. F. Brown, in Cincinnati Medical Almanac.*

